# Polymeric Nanomaterials for Nanotherapeutics. Ed. C. Vasile, Elsevier, 1st Edition. 2018. Series Volume Editors: Cornelia Vasile, eBook ISBN: 9780128139332; Paperback ISBN: 9780128139325: Elsevier, Published Date: 2nd November 2018; 558 pages. In Micro and Nano Technologies Series

**DOI:** 10.3390/polym11091452

**Published:** 2019-09-04

**Authors:** Marek Kozlowski

**Affiliations:** Wroclaw University of Science and Technology, 50-370 Wrocław, Poland; marek.a.kozlowski@pwr.edu.pl

In the book “**Polymeric Nanomaterials in Nanotherapeutics**” (2018) edited by **prof. Dr. Cornelia Vasile**, the authors show how polymeric nanomaterials are used in new emerging fields of medicine, namely nanomedicine and nanotherapeutics. The book content covers the whole field of nanomedicine, from definition to applications and recent trends. The readers will discover, from the book’s various chapters, how polymeric nanomaterials are used to obtain nanosensors and nanorobots as the biomedical instrumentation, surgery, diagnosis and controlled/targeted drug delivery for cancer therapy, improved pharmacokinetics, monitoring of diabetes and other healthcare problems. The polymeric nanomaterials described in this book are classified by several criteria such as their origin (natural and/or synthetic), structure, morphology, form and applications, e.g., polymeric nanoparticles, micelles, nanogels, nanoemulsions, dendrimers, polymers as drugs, polymer-drug conjugates, polyplexes, multifunctional nanoparticles, polymersomes, stimuli-responsive nanomaterials, etc. Moreover, the advancements in nanotherapeutics are described, which are related to development of new drug formulations based on nanomaterials and elaborating various routes of administration for controlled and targeted delivery and release of active agents to non-healthy tissues and cells. Topics worthy of particular discussion are: Responsive polymeric nanotherapeutics, Nanomaterials derived from phosphorus-containing polymers: diversity of structures and applications, Nucleic acids based bionanomaterials for drugs and gene therapy, Electrospun polymeric nanostructures with applications in nanomedicine, nanocoatings: preparation, properties, and biomedical applications, Functionalization of polymer materials for medical applications using chitosan nanolayers, Biological applications of nanoparticles in optical microscopy, Regulatory status of therapeutic polymeric nanomaterials. 

The book is structured in 15 chapters that are co-authored by various specialists in the field of polymeric nanomaterials and pharmacy from which I can mention prof. dr. Lidija Fras Zemljic (University of Maribor, Slovenia), dr. Anca Margineanu (Max Delbruck Centrum, Germany), dr. Neli Koseva (Institute of Polymers, Bulgarian Academy of Sciences, Bulgaria), prof. dr. Onur Yilmaz (Ege University, Turkey), prof. dr. Mihaela C. Baican and prof. dr. Lenuta Profire (“Grigore T. Popa” University of Medicine and Pharmacy, Romania), dr. Aurica P. Chiriac (“Petru Poni” Institute of Macromolecular Chemistry, Romania). Going through the chapters of the book, the reader will learn about the ways of administering nanotherapeutics (oral, dental, topical and transdermal, pulmonary and nasal, ocular, vaginal, and brain drug delivery) and how these nanotherapies are applied to dental work, wound healing, cancer, cardiovascular diseases, neurodegenerative disorders, infectious diseases, chronic inflammatory diseases, metabolic diseases, and so on. 

Moreover, there are presented various development stages of new drugs containing polymeric nanomaterials as carriers (research level, clinical trial, commercial stage, etc.) and if they are approved by U.S. Food and Drug Administration (FDA) or European Medicines Agency (EMA). In all chapters the aspects related to the risk assessment for the presented nanotherapeutics, by toxicity evaluation and regulatory status, are highlighted.

The book is distinguished by the following specific features: it presents how polymeric nanomaterials are used to develop and create efficient therapies/medical treatments; the manner in which these polymers are applied in different biomedical areas (disease prevention, diagnosis, drug development, and improvement of existing treatments), focusing on highlighting the advantages of nanotherapeutics. The book has been written in a manner accessible for scholars, researchers, postgraduate students and specialists in the fields of nanomaterials, polymeric bionanomaterials, biotechnology, and in biomedicine. 

